# Administration of progesterone on the day of oocyte retrieval to reverse accumulation of fluid in the endometrial cavity during assisted reproductive techniques

**DOI:** 10.5935/1518-0557.20140019

**Published:** 2014

**Authors:** Mario Cavagna, Claudia G. Petersen, Ana L. Mauri, João Batista A. Oliveira, Ricardo R.L. Baruffi, José G. Franco Jr.

**Affiliations:** 1 Center for Human Reproduction Prof. Franco Jr, Ribeirao Preto, SP, Brazil; 2 Paulista Center for Diagnosis Research and Training, Ribeirao Preto, SP, Brazil; 3 Women’s Health Reference Centre, Perola Byington Hospital, Sao Paulo, SP, Brazil.

**Keywords:** Deeply infiltrating endometriosis, chronic pelvic pain, infertility, surgical complications, laparoscopy

## Abstract

**Objective:**

To determine whether administration of progesterone on the day of oocyte retrieval may reverse accumulation of fluid in the endometrial cavity.

**Methods:**

A total of 50 patients who underwent assisted reproductive technology (ART) cycles with endometrial cavity fluid (ECF) observed by ultrasound at the time of oocyte retrieval were included. Upon the identification of ECF, vaginal administration of natural progesterone was started. Two days later, the endometrial cavity was re-evaluated, and embryo transfer was performed in the absence of ECF.

**Results:**

ECF was absent two days after administration of vaginal progesterone in 47 of the 50 patients (94%). ECF persisted in 3 of the 50 patients (6%). The clinical pregnancy rate per transfer was 34.0%, and the implantation rate was 21.6%.

**Conclusion:**

Our data suggest that, in the presence of ECF, administration of intravaginal progesterone in ART cycles must be initiated on the day of follicle aspiration to reverse ECF and to avoid the deleterious effects of fluid on the blastocyst-endometrial interaction.

## INTRODUCTION

The main factors responsible for a successful embryonic implantation are the embryo quality and endometrial receptivity. An adequate interaction and synchrony between the endometrium and the blastocyst are required for the implantation process (Cavagna *et al.*, 2006; Edgell *et al.*, 2013). Given that, it is important to perform embryo transfer in assisted reproductive technique (ART) cycles in the presence of optimal conditions of endometrial receptivity. One of the possible mechanisms of impaired endometrial receptivity is the presence of endometrial cavity fluid (ECF) on the day of embryo transfer (Sharara & McClamrock, 1997; Sharara & Prough, 1998; Levi *et al.*, 2001). Although accumulation of fluid within the endometrial cavity is uncommon during ART, it is detrimental to embryonic implantation and negatively affects outcomes, as embryonic apposition is impaired if a fluid layer is overlaying the endometrium. The incidence of ECF in ART cycles is approximately 3%, and the outcome is particularly negative when the ECF is >3.5 mm in antero-posterior diameter (He *et al.*, 2010). Tubal infertility, polycystic ovary syndrome (PCOS), poor ovarian response, subclinical uterine infections and physiological production in the genital tract are associated with the presence of ECF (Levi *et al.*, 2001; Griffiths *et al.*, 2002). Akman *et al.* (2005) have shown that it is more common to see fluid accumulation inside the endometrium in ART cycles of PCOS patients than tubal factor patients (22.3% versus 11.1%). However, the exact mechanism of ECF generation remains unclear, and optimal treatments for ECF have not been determined. The aim of this investigation is to determine whether therapeutic doses of natural vaginal progesterone, beginning on the day of oocyte retrieval, can reverse accumulation of fluid in the endometrial cavity and provide satisfactory clinical pregnancy rates.

## MATERIAL AND METHODS

**Figure 1:**
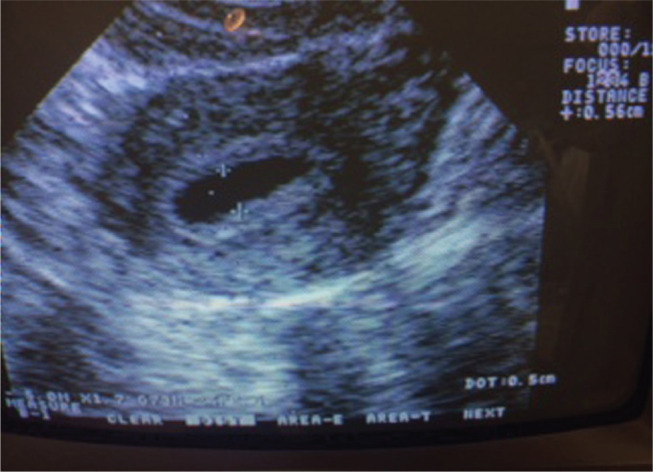
Endometrial cavity fluid (ECF). The day of oocyte retrieval. Fluid accumulation in the uterine cavity detected by transvaginal ultrasound in a sagittal view (A-P diameter 5.6 mm).

A prospective cohort study was conducted from March 2011 to November 2013 at the Center for Human Reproduction Prof. Franco Jr., Ribeirão Preto, Brazil. A total of 50 patients who underwent ART cycles with ECF observed by ultrasound at the time of oocyte retrieval (Fig 1) were included (4.9% of the total of IVF/ICS cycles in this period). The mean fluid accumulation in the uterine cavity detected by transvaginal ultrasound in a sagittal view (A-P diameter) was 3.0±1.8 mm (2-7.8).

The epidemiological data for the patients are presented in table 1 (age, diagnosis, number of total and mature oocytes, etc.) In all cases, ovarian stimulation was performed with recombinant FSH (150-300 IU daily) and recombinant LH (75-150 IU daily). Pituitary suppression was carried out either with agonists (long protocol: 66%) or antagonists (multi doses: 34%) of GnRH. All patients were given 250 µg recombinant hCG for triggering ovulation, and oocyte retrieval was carried out 36 hours later. Upon the identification of ECF, vaginal administration of natural progesterone at a total dose of 180 mg/day (Crinone^®^ 8% - Serono - Brazil, twice daily - 90 mg of natural progesterone in each vaginal application) was started (Day 0). Two days later (Day 2), the endometrial cavity was re-assessed. It should be stressed that progesterone is started routinely in our Clinic only on the day of embryo transfer (Baruffi *et al.*, 2003), carried out on Day 2. If ECF was absent, embryo transfer was performed. If ECF persisted, the embryos were frozen.

**Table I:** General characteristics of the study population

Characteristic	Total	After Progesterone administration		
		Absence of ECF	Persistence of ECF	*P*
Patients	50	47	3	
Cycles	50	47	3	
Age (years)	33.9±4.8 (21-43)	34.1±4.6 (21-43)	30.0±8.1 (23-39)	0.48
BMI	23.9±3.7	24.0±3.8	22.0±0.2	0.45
Etiology Idiopathic Male Endometriosis Tuboperitoneal Tuboperitoneal + endometriosis	30.0% (15/50) 36.0% (18/50) 20.0% (10/50) 10.0% (05/50) 4.0% (02/50)	29.8% (14/47) 36.2% (17/47) 21.3% (10/47) 8.5% (04/47) 4.2% (02/47)	33.3% (1/3) 33.3% (1/3) 0 33.3% (1/3) 0	0.66
Fluid accumulation Sagittal view (A-P diameter)	3.0±1.8 mm (2-7.8)	3.0±1.7 mm (2-7.8)	3.0±1.3 mm (2-5.6)	0.75
Oocytes (n) Metaphase II Total	8.2±6.0 11.8±7.9	7.7 ± 5.5 11.2±7.5	12.7±9.7 16.0±12.2	0.40 0.63
Fertilization rate	70.8±22.5%	71.1±23.3	68.4±16.6	0.71
Number of embryos	5.6±3.7	5.4±3.4	7.7±6.5	0.60
Embryo transferred (n)	2.1±0.6	2.1±0.6	----	
Pregnancy rate/transfer	34%(16/47)	34%(16/47)	----	
Miscarriage rate	6.3%(1/16)	6.3%(1/16)	----	

ECF: endometrial cavity fluid

## RESULTS

ECF was absent two days after administration of vaginal progesterone in 47 of the 50 patients (94.0%). ECF persisted in 3 of the 50 patients (6.0%). There were no significant differences with regard to age (*P*=0.48), etiology (*P*=0.66), number of oocytes collected (*P*=0.63), mature oocytes *(P*=0.40) or embryos (*P*=0.60) between the groups with absence or persistence of ECF after vaginal progesterone administration.

ECF persisted after the use of progesterone for the only patient with hydrosalpinx. There were three patients with PCOS in the group without ECF and two patients with PCOS in the group in which ECF persisted after administration of progesterone. In all patients, 97 embryos were transferred (2.1 per patient) and 21 had successful implantation (implantation rate: 21.6%). The clinical pregnancy rate was 32% (16/50) per patient and 34% (16/47) per transfer. There was only one miscarriage.

## DISCUSSION

Endometrial receptivity is a major issue affecting implantation rates in assisted reproductive techniques (ART). There is consensus that, in ART cycles, luteal phase support with progesterone is mandatory for the promotion of an adequate environment, which in turn improves implantation rates and permits the development of the transferred embryos. Ovarian stimulation during ART cycles, as well as the utilization of GnRH analogs and follicle aspiration, lead to an endocrine disturbance during the luteal phase, interfering with the production of progesterone (Gazvani *et al.*, 2012). Intravaginal progesterone administration is well tolerated and was demonstrated to be even more efficient than intramuscular injection, so that it remains the preferred route of administration (Smitz *et al.*, 1992).

Regarding the duration of the supplementation, progesterone has been used for as little as two weeks and for as long as 12 weeks of gestation (Gazvani *et al.*, 2012). With regard of the beginning of luteal support, while most infertility centers initiate progesterone on the day of oocyte retrieval, in approximately 15% of the cycles progesterone administration is initiated on the day of embryo transfer (Vaisbuch *et al.*, 2014).

ECF is a fluid accumulation within the endometrial cavity, which is related to poor outcomes in ART cycles (He *et al.*, 2010) In the presence of ECF, the relevant treatment options include expectant treatment, postponing embryo transfer, and transvaginal sonographic ECF aspiration (He & Zhu, 2011). Given that ECF impairs the implantation process, cryopreservation of all embryos and subsequent transfer was considered the best approach (Nikolettos *et al.*, 2002).

This investigation was designed to determine whether initiating progesterone supplementation on the day of oocyte retrieval could reverse accumulation of fluid within the endometrial cavity, thus allowing embryo transfer in the same ART cycle. Our results indicated that 47 of 50 patients (94.0%) were negative for ECF two days after administration of intravaginal progesterone. The procedure effectively reduced the ECF visualized by ultrasound, and as such should be recommended as the main option for management of ECF in ART cycles. The mechanism by which progesterone may reverse ECF is not clear. Progesterone has anti-angiogenic effects and inhibits endothelial cell proliferation through a nuclear receptor-mediated mechanism (Hsu *et al.*, 2008) and may reduce proliferation of endometrial stromal cells and suppress the transcription of VEGF (Rocha *et al.*, 2013). These facts may exert a role in the disappearance of ECF with early administration of progesterone. Anti-inflammatory effects of progesterone, which make possible its use in neurologic situations, such as spinal cord contusions and other neurological disorders (De Nicola *et al.*, 2013; Garcia-Ovejero *et al.*, 2014), may also be responsible for the reduction of ECF. Moreover, progesterone regulates decidual prolactin expression, which is a marker of decidualization, and thus exerts a role in differentiating human endometrial stromal cells (Brosens *et al.*, 1999), The effects of progesterone on differentiation of the endometrium and its anti-inflammatory and anti-angiogenic activities may explain the suppression of ECF after the treatment.

Despite recruiting all eligible participants during the study period, the sample size was small, and spontaneous remission may have occurred in some of the patients. Although spontaneous remission may have occurred, the positive results (i.e., absence of ECF in 94.0% of patients) in this observational study demonstrate that administration of vaginal progesterone beginning on the day of oocyte retrieval is an acceptable option for managing ECF in women undergoing assisted reproduction.

In conclusion, our data suggest that, in the presence of ECF, administration of intravaginal progesterone in ART cycles must be initiated on the day of follicle aspiration to reverse ECF and avoid the deleterious effects of fluid on blastocyst-endometrial interactions.
